# Exploratory Statistical Analyses of Clinical and Biochemical Factors for Differentiated Thyroid Cancer from a Romanian Cohort

**DOI:** 10.3390/cancers18061036

**Published:** 2026-03-23

**Authors:** Alexandru Dima, Irina-Oana Lixandru-Petre, Denis Iorga, Gratiela Gradisteanu Pircalabioru, Dana Cristina Terzea, Andrei Goldstein, Florina Silvia Iliescu, Mihai Dascalu, Madalina Musat, Ciprian Iliescu

**Affiliations:** 1 Faculty of Automatic Control and Computer Science, National University of Science and Technology POLITEHNICA Bucharest, 060042 Bucharest, Romania; 2 Academy of Romanian Scientists, Ilfov 3, 050044 Bucharest, Romania; 3 eBio-Hub Centre of Excellence in Bioengineering, National University of Science and Technology POLITEHNICA Bucharest, 060042 Bucharest, Romania; 4 Department of Botany and Microbiology, Faculty of Biology, University of Bucharest, 050095 Bucharest, Romania; 5 C.I. Parhon National Institute of Endocrinology, 011863 Bucharest, Romania; 6 Faculty of Material Science and Engineering, National University of Science and Technology POLITEHNICA Bucharest, 060042 Bucharest, Romania; 7 National Institute for Research and Development in Microtechnology-IMT Bucharest, 077190 Bucharest, Romania; 8 Department of Endocrinology, Carol Davila University of Medicine and Pharmacy, 020021 Bucharest, Romania

**Keywords:** thyroid cancer, statistical correlations, electronic medical records, clinical data

## Abstract

Background/Objectives: Thyroid cancer (TC) is among the most common endocrine malignancies, with incidence rates increasing worldwide. However, careful inferential analysis based on refined data is needed to provide a sharper clinical and epidemiological description of this serious condition in a biologically and technologically evolving society. This study presents an exploratory statistical analysis of data from 1470 patients who underwent thyroid surgery for differentiated TC. Methods: The analysis combines bivariate exploration of associations between variables with univariate and multivariate analyses stratified by histological subtype. We examined pathological characteristics (tumor location, nodal metastases, distant metastases, margin involvement, lymphovascular invasion, vascular invasion, and perineural invasion), clinical characteristics (clinical stage), biochemical markers (thyroglobulin, anti-thyroglobulin antibodies, and thyroid-stimulating hormone), and demographic variables (sex assigned at birth and age). In addition, exploratory multivariable models were used to investigate factors associated with lymph node metastasis and margin involvement in papillary microcarcinoma, the diffuse sclerosing variant, and the classical variant of papillary thyroid carcinoma. Results: Notably, moderate to high effect size correlations highlight the interdependence of invasive histopathological features in thyroid cancer and their collective link to adverse surgical outcomes and prognosis. Conclusions: This study provides an analysis of associations between the variables and subtype-specific descriptive estimates, serving as a foundation for future work in tailoring personalized medicine.

## Simple Summary

Thyroid cancer is one of the most common endocrine cancers, and its incidence has been increasing worldwide. Understanding how different clinical, pathological, and biochemical factors are related may help improve patient management and follow-up strategies. In this study, we analyzed data from 1470 patients who underwent thyroid surgery for differentiated thyroid cancer. We examined relationships between tumor characteristics, clinical stage, biochemical markers, and demographic factors, with particular attention to different histological subtypes. Our analysis revealed meaningful associations between several invasive tumor features and indicators of disease progression. These findings contribute to a better understanding of how multiple factors interact in thyroid cancer and provide a foundation for future research aimed at improving risk assessment and personalized treatment approaches.

## 1. Introduction

Thyroid cancer is the most common malignant lesion of the endocrine glands [1]. The increase in thyroid cancer incidence observed between 1974 and 2013 affected all stages of disease, while the mortality among patients with advanced thyroid cancer rose by approximately 1.1% per year [2]. Notably, the European Network of Cancer Registries (ENCR) (https://www.encr.eu/sites/default/files/factsheets/ENCR_Factsheet_Thyroid_2017-2.pdf, accessed on 19 March 2026) reported an average age-standardized incidence rate of approximately 6.3 per 100,000 person-years for thyroid cancer (TC) overall, with differentiated thyroid cancer (DTC) being the most prevalent. According to the World Health Organization’s (WHO) latest classification of thyroid tumors [3], DTC includes manifestations as papillary thyroid carcinoma (PTC), follicular thyroid carcinoma (FTC), and oncocytic thyroid carcinoma (OTC), comprising over >90% in all thyroid cancers [4]. Other, less common but still relevant types include thyroid C-cell-derived carcinomas, such as medullary thyroid carcinoma (MTC).

Moreover, the increasing incidence of differentiated thyroid carcinoma in children and adolescents has become a growing concern [5]. DTC is also among the most frequently diagnosed cancers in the United States, where estimates showed 44,020 new cases of thyroid cancer in 2024 [6] compared with 37,200 in 2015 when the last American Thyroid Association (ATA) guidelines were published. Other sources reported that the yearly incidence increased threefold from 1975 (4.9 per 100,000) to 2015 (14.3 per 100,000) [7] and that the tumors less than 1 cm were around 25% of the cases diagnosed between 1988 and 1989, while those between 2008–2009 were 39% [7]. Interestingly, while the incidence of differentiated thyroid cancer has increased, thyroid cancer cases in general, and particularly small thyroid cancers, have shown reduced incidence in the United States since 2014 [6]. However, an analysis derived from the Global Burden of Disease Study 2021 showed a 55% increase in prevalence, a 7% decrease in mortality, and a 4.2% reduction in age-standardized rates since 1990 [8].

The change in the data could reflect the shift to earlier detection and diagnosis, correlated with the increasing use of neck ultrasonography, ultrasound-guided fine-needle aspiration, and other modern imaging modalities [9]. Moreover, improvements in diagnostic modalities have been associated with increased detection and reported incidence of DTC over the past three decades, while advances in treatment have contributed to stable disease-specific mortality. While most patients have an excellent prognosis, the increasing diagnoses created a vast database of thyroid cancer survivors who require surveillance, engaging more responsibilities. Here is where diagnosis intersects with overdiagnosis, a risk recognized almost 30 years ago [10] and commonly observed in the USA and Europe [11]. It is already acknowledged that epidemiology supports therapeutic and health policy approaches based on various risk factors.

Statistical insights from DTC cohorts established risk factors for thyroid cancer, such as exposure to ionizing radiation (e.g., environmental irradiation during childhood or adolescence, head and neck during childhood, total body irradiation for bone marrow transplantation) [12], and familial inherited specific genetic mutations (e.g., BRAF V600E that predict progression and guide treatment) [13]. Data also indicated that risk factors such as higher body mass index [14], certain reproductive or hormonal factors (e.g., younger menarche, hormone replacement therapy) [15], potentially elevated thyroid-stimulating hormone (TSH) (without autoimmunity) [16], or exposure to polybrominated diphenyl ethers and organophosphate flame [17] correlated with more aggressive features (e.g., larger multifocal tumours, nodal metastasis) or higher risk classifications [18].

Interestingly, after a steady rise spanning approximately 30 years, the incidence of thyroid cancer reached its highest level in 2015, at 14.9 cases per 100,000 population, followed by a subsequent reduction in incidence between 2015 and 2017, because of a change in the routine clinical practice of screening for thyroid cancer to avoid overdiagnosis [2]. The earlier increase was attributed to a genuine rise in papillary thyroid carcinoma and to intensified detection resulting from widespread use of neck imaging and fine-needle aspiration of thyroid nodules, leading to concerns about overdiagnosis. Overdiagnosis refers to the identification of malignancies that would not have caused clinical symptoms or mortality if left untreated.

Therefore, models such as the DATA model in the ATA guidelines [2] are essential. This model applies from initial cancer diagnosis through the detection of residual disease or clinical recurrence. It emphasizes a careful evaluation of whether an intervention is warranted by weighing potential benefits against risks, while also incorporating patient-specific factors and preferences. Models are crucial for disease management, as they provide structured frameworks for clinical decision-making that support both patients and clinicians throughout the course of thyroid cancer, ultimately improving quality of life and health economics in an era of personalized, AI-driven medicine [19]. Comprehensive epidemiological data are used to attentively evaluate the potential benefits of treatment against the associated risks, because therapeutic interventions may sometimes impose a greater burden than the disease itself. Moreover, previous data support tumor staging models for estimating disease-specific mortality risk and enable initial risk stratification to predict the likelihood of disease persistence or recurrence over both the short and long term.

Over the past two to three decades, substantial progress has been made in understanding differentiated thyroid cancer, resulting in important improvements in both diagnostic strategies and therapeutic options. DTC generally has a good prognosis after thyroidectomy, with over 90% survival at 20 years after diagnosis in PTC. Postoperative treatment includes thyroid hormone replacement to suppress TSH and, in selected cases with intermediate or high recurrence risk, radioactive iodine administration. Life-long follow-ups for tumor markers (thyroglobulin, antithyroglobulin antibodies), ultrasound, and scintigraphy evaluations remain the norm.

Despite these advances and existing guidelines, significant debate remains in several aspects of clinical management. Ongoing controversies, variations in how evidence is interpreted, differences in clinical practice across regions and specialties, and unequal access to diagnostic tests and approved therapies among countries all contribute to this heterogeneity. The complexity of patient selection for radioiodine therapy, together with continuously evolving clinical guidelines, has led to discrepancies in clinical practice. These discrepancies underscore the need for further research to address areas of uncertainty and to strengthen evidence-based guidance for individualized patient care.

Therefore, the present study retrospectively analyzes a newly introduced dataset of 1556 patients diagnosed with differentiated thyroid cancer who underwent surgical tumor removal followed by radioiodine therapy; out of this sample, 1470 records contained fully evaluable patient data. The dataset enables the investigation of correlations among multiple clinicopathological variables relevant to treatment decision-making.

The primary objective of this study is to explore potential associations among clinical, pathological, biochemical, and demographic variables in a cohort of cancer patients. Rather than testing a predefined hypothesis, the analysis aimed to identify patterns within the dataset that could generate hypotheses for future research. Given the exploratory nature of the analysis and the large number of statistical tests performed, the findings should be interpreted as hypothesis-generating rather than confirmatory. A secondary objective is to conduct subtype-specific analyses to examine associations within individual subtypes and provide descriptive estimates of these variables at the subtype level.

Accordingly, the primary research question is: “What patterns of association among clinical, pathological, biochemical, and demographic variables can be identified?” Secondary research questions are: “What patterns of association can be identified within individual thyroid cancer subtypes?” and “How do thyroid cancer subtypes differ with respect to these characteristics?”

## 2. Method

### 2.1. Dataset Creation and Preprocessing

Data records from a cohort of 1556 adult patients diagnosed with DTC and treated at the Romanian National Institute of Endocrinology “C.I. Parhon” (CIPNIE) between 2022 and 2024 were collected and analyzed in this study. The study population comprised 1233 female and 323 male patients, with 1458 cases of papillary carcinoma, 73 cases of follicular carcinoma, and 25 cases of mixed histology. The cohort predominantly consisted of papillary thyroid carcinoma cases, reflecting the epidemiological distribution of thyroid cancer within the institutional caseload. Small numbers of follicular thyroid carcinoma and mixed histology tumors were also present in the database and were retained to reflect the real-world distribution of cases treated during the study period.

All patients involved in the study underwent total or near-total thyroidectomy followed by radioiodine therapy. The cohort was restricted to patients who underwent total or near-total thyroidectomy followed by radioiodine therapy to obtain a clinically homogeneous population with an intermediate-to-high risk of recurrence requiring adjuvant treatment. At the host institution, patients receiving radioiodine underwent standardized postoperative assessment, including biochemical monitoring (thyroglobulin and anti-thyroglobulin antibodies) and whole-body scintigraphy, ensuring consistent follow-up data across the cohort and enabling reliable clinicopathological correlations relevant to radioiodine dosimetry and follow-up strategies.

The study protocol was approved by the Ethics Committee of the Scientific Council of CIPNIE before accessing the medical records. Data collection was conducted in accordance with the General Data Protection Regulation (GDPR) and applicable national legislation, ensuring anonymization of sensitive information and adherence to confidentiality agreements by all investigators involved in data handling. The collected dataset integrates an extensive set of variables extracted from the institutional Hipocrate database, specifically from both the structured data fields and the narrative components of each patient’s Electronic Health Record (EHR).

The extracted variables encompassed multiple clinical, pathological, biochemical, and therapeutic domains. Demographic and baseline clinical data included patient age at the time of thyroid surgery and sex assigned at birth. Tumor-related characteristics comprised the anatomical location of the primary lesion (left or right thyroid lobe, isthmus, or pyramidal lobe), histopathological diagnosis (papillary, follicular, or mixed thyroid carcinoma), and histological subtype when specified in the pathology report (e.g., classic, follicular variant, oncocytic variant, or other subtypes).

Disease extent was documented using the 8^th^ edition of the TNM classification of malignant tumors system [20], including primary tumor stage (T1–T4 or Tx), nodal status (pN0, pN1, or pNx), and distant metastasis status (M0, M1, or Mx). Overall clinical stage (ST1–ST4 or STx) was defined according to patient age and aggregated TNM findings. Additional pathological parameters analyzed included tumor margin involvement (R0 refers to clean, cancer-free margin of the resected specimen; R1 combines both microscopic cancer cells present at the inked edge (R1) and macroscopic tumor margin involvement (R2); Rx: indeterminate), the presence or absence of vascular invasion (V), lymphovascular invasion (LV), and perineural invasion (PNI), each coded as present, absent, or indeterminate.

Postoperative biochemical markers obtained before the first radioiodine therapy session included stimulated thyroglobulin (Tg, ng/mL), anti-thyroglobulin antibodies (Anti-TG, UI/mL), and thyroid-stimulating hormone (TSH, µUI/mL). Imaging and follow-up assessments comprised postoperative neck ultrasound and whole-body scintigraphy (WBS). Therapeutic data included surgical removal of the primary tumor as initial treatment, the total cumulative activity of administered radioiodine (I-131), fractionated doses, and the number of radioiodine therapy sessions.

From a data-processing perspective, two fields (i.e., “cancer type” and “location”) exhibited a multi-label structure, as a single patient could have multiple cancer types or tumor locations simultaneously. To integrate these variables into downstream statistical analyses, we applied a binarization procedure to convert each distinct label into a separate indicator variable.

The cancer subtype variable was derived by consolidating the original histopathological subtype annotations. All mixed variants were grouped into a single category labeled “Mixed variant of PTC/FTC”, while all remaining subtypes with fewer than 10 occurrences were combined into a “Rare PTC/FTC variants (n < 10)” category, where n is the number of cases. The threshold of 10 cases was selected to limit category sparsity, ensure the validity of contingency-based statistical tests, and reduce instability in effect size estimation associated with very low cell counts.

The missing-data assessment revealed incomplete entries across several variables, largely due to incomplete documentation in the EHRs. We adopted an exclusion criterion focused on essential biochemical variables. Records with missing values for TG, anti-TG, or TSH were excluded, as these measurements constitute core variables required to accurately characterize patients’ post-surgical and post-radioiodine therapy status. According to the study protocol, these laboratory values were expected to be recorded for all patients after the first radioiodine treatment. Therefore, their absence suggested that the data collection protocol had not been fully adhered to in those cases. Because the reliability and completeness of the remaining information in such records could not be ensured, these cases were removed from the analysis. After applying the filters, the working dataset used in subsequent analyses comprised 1470 fully evaluable patient records. A full breakdown of the statistics of our data is available in Table 1 and Table 2.

### 2.2. Bivariate Analysis

We examined the dataset to identify both unanticipated associations among variables and evaluate whether theoretically anticipated relationships could be empirically substantiated. Because the dataset contains a mixture of categorical and quantitative variables, the analysis encompasses three classes of pairwise relationships: categorical–categorical, quantitative–quantitative, and categorical–quantitative. For each class, an appropriate statistical procedure was selected to evaluate the presence and strength of association. Non-parametric methods were preferred to avoid distributional assumptions.

We complemented each hypothesis test with an effect size estimate, enabling us to evaluate the statistical significance and the substantive magnitude of observed associations. The methodological approach for each relationship type is summarized below:

1. Categorical–Categorical: Associations between categorical variables were evaluated using Fisher’s Exact Test [21], which is appropriate for contingency tables with small or uneven cell counts and does not rely on asymptotic approximations. To quantify the strength of association independently of table size, we computed Cramer’s V [22], which provides a standardized effect size ranging from 0 (no association) to 1 (perfect association).
2. Quantitative–Quantitative: To assess relationships between pairs of quantitative variables, we computed both Pearson’s product–moment correlation coefficient [23] and Spearman’s rank correlation coefficient [24]. Pearson’s correlation was used to evaluate linear associations between approximately normally distributed variables. In contrast, Spearman’s correlation provided a nonparametric measure of monotonic association that is robust to non-normality and outliers. Each correlation coefficient inherently serves as an effect size measure, enabling direct interpretation of the strength of the association.
3. Categorical–Quantitative: For comparisons involving a quantitative variable and a categorical variable with more than two groups, the Kruskal–Wallis test [25] was employed. This is a non-parametric alternative to one-way ANOVA that operates on ranked data. Effect size was quantified using epsilon-squared, calculated from the ANOVA on ranks, providing an estimate of the proportion of variability attributable to group differences. When the categorical variable was binary, we used the Mann–Whitney U test [26], a non-parametric alternative to the two-sample *t*-test. Effect size was expressed via the rank-biserial correlation, which captures the degree of separation between the two distributions and is readily interpretable.

Given the large number of pairwise comparisons performed, statistically significant results were expected to arise by chance alone. To mitigate this risk, we corrected all *p*-values (which quantify the probability of observing results at least as extreme as those obtained under the null hypothesis) for multiple comparisons using the False Discovery Rate (FDR) procedure of Benjamini and Hochberg [27]. This approach controls the expected proportion of false positives among rejected hypotheses while retaining greater statistical power than more conservative methods, such as the Bonferroni correction. Only the FDR-adjusted *p*-values are reported in the results, and statistical significance is defined as an adjusted *p*-value of 0.05 or lower.

Missing data patterns varied across pairs of variables, resulting in different sample sizes for different tests. Consequently, reliance solely on *p*-values may be misleading, as significance can be influenced by sample size rather than by the substantive strength of an association. In addition to statistical significance, we examined the effect size for each test. Together, the *p*-values and effect sizes created a four-tier interpretation framework:

(a) The association is not statistically significant (adjusted *p* > 0.05);
(b) The association is statistically significant, but the effect size is small;
(c) The association is statistically significant with a moderate effect size;
(d) The association is statistically significant with a large effect size.

Effect sizes were interpreted using established, metric-specific thresholds appropriate for each statistical test.

For correlations between numerical variables, correlation coefficients (r) were interpreted as effect sizes using commonly applied thresholds (e.g., <0.10 negligible, 0.10–0.39 weak, 0.40–0.69 moderate, 0.70–0.89 strong, ≥0.90 very strong), while acknowledging that such interpretations can be context-dependent and not universally applicable [28].

For associations between categorical variables, effect size was quantified using Cramer’s V. Interpretation thresholds were adapted based on the dimensionality of the contingency table, following established recommendations: for 2×2 tables, values of <0.30, 0.30–0.49, and ≥0.50 were considered weak, moderate, and strong, respectively; for 3×3 tables, <0.20, 0.20–0.34, and ≥0.35; and for larger tables, <0.17, 0.17–0.28, and ≥0.29 [29].

For comparisons between numerical and categorical variables, effect sizes were expressed using rank-biserial correlation (for Mann–Whitney tests) and eta-squared ($\eta^{2}$) for Kruskal–Wallis tests. Rank-biserial correlation values of <0.28, 0.28–0.42, and ≥0.43 were considered small, medium, and large, respectively, while $\eta^{2}$ values of <0.08, 0.08–0.25, and ≥0.26 were interpreted as small, medium, and large effects [30,31].

To support the interpretation of the correlation analyses and characterize the underlying data structure, we also used a series of descriptive visualizations tailored to the types of variables examined: violin plots for categorical-quantitative relationships, and heatmaps for categorical–categorical relationships. All heatmaps presented in this study are row-normalized, meaning that values within each row are scaled to a 0–1 range. This allows color intensity to reflect relative differences within each variable, improving comparability across variables with different value ranges. The corresponding unnormalized heatmaps, which preserve absolute values, are provided in the Supplementary Material (heatmaps_unnormalized.docx) for completeness and to allow direct assessment of the original data distribution.

### 2.3. Subtype Analysis

Furthermore, we examined differences between cancer subtypes. For this analysis, we excluded cases marked as uncertain regarding cancer subtype (n = 151). The subtype analysis first focused on identifying significant associations between cancer subtype and other variables in the dataset. Methods similar to those used in the bivariate analysis were applied.

Next, estimates and corresponding confidence intervals for histological and biological variables were produced stratified by phenotype. Confidence intervals for proportions were computed using the Wilson method, while confidence intervals for means were computed using a t-distribution-based method.

The Section 3 provides a narrative description of findings from cancer subtype analyses, including selected estimates for the most prevalent subtypes, while additional analyses and heatmaps are reported in the Supplementary Material.

Additionally, we conducted an exploratory subtype-stratified multivariable analysis using the generalized linear model framework [32] to explore clinicopathological associations. Specifically, for the most prevalent subtypes (papillary microcarcinoma, diffuse sclerosing variant of PTC, classical variant of PTC), we fitted generalized linear models with a binomial family and logit link (i.e., logistic regression) to model lymph node metastasis and margin involvement.

Model specification was deliberately restricted to a reduced set of predictors reflecting tumor burden (tumor size category), patient-related factors (age and sex assigned at birth), anatomically distinct tumor location (isthmus involvement), and invasive growth patterns (lymphovascular invasion). This model specification was adopted to ensure identifiability and stability within subtype strata, as including additional histopathological variables yielded non-identifiable estimates due to complete or quasi-complete separation.

To model lymph node metastasis (N), we constructed a multivariable regression model including primary tumor stage (T), patient age, sex, isthmus tumor location (ISTM), and lymphovascular invasion (LV) as predictors (“N ∼ T + Age + Sex + ISTM + LV”). A separate multivariable model was used to evaluate margin involvement (R), with primary tumor stage (T), lymph node status (N), age, sex, isthmus tumor location (ISTM), and lymphovascular invasion (LV) included as explanatory variables (“R ∼ T + N + Age + Sex + ISTM + LV”). The same structure was applied across the three subtypes (PC, diffuse PTC, PTC) to facilitate direct comparison of effect patterns and to assess shared versus subtype-specific relationships. Each model was applied to a different sample size due to missing data. Model diagnostics were performed to assess the influence of extreme values and overall fit.

## 3. Results

A total of 195 statistical tests were conducted, of which 70 remained statistically significant after controlling for multiple comparisons using the false discovery rate (FDR) procedure. Given the relatively large number of significant associations identified, subsequent reporting primarily emphasized correlations exhibiting at least a moderate effect size, as these are more likely to be of practical and biological relevance. Nevertheless, a small number of statistically significant associations with small effect sizes are also reported. These associations should be interpreted with caution, as their statistical significance may primarily reflect the large sample size rather than a biologically or clinically meaningful relationship. Consequently, these findings are presented primarily as exploratory observations that may inform hypotheses for future research.

**Figure 1:**
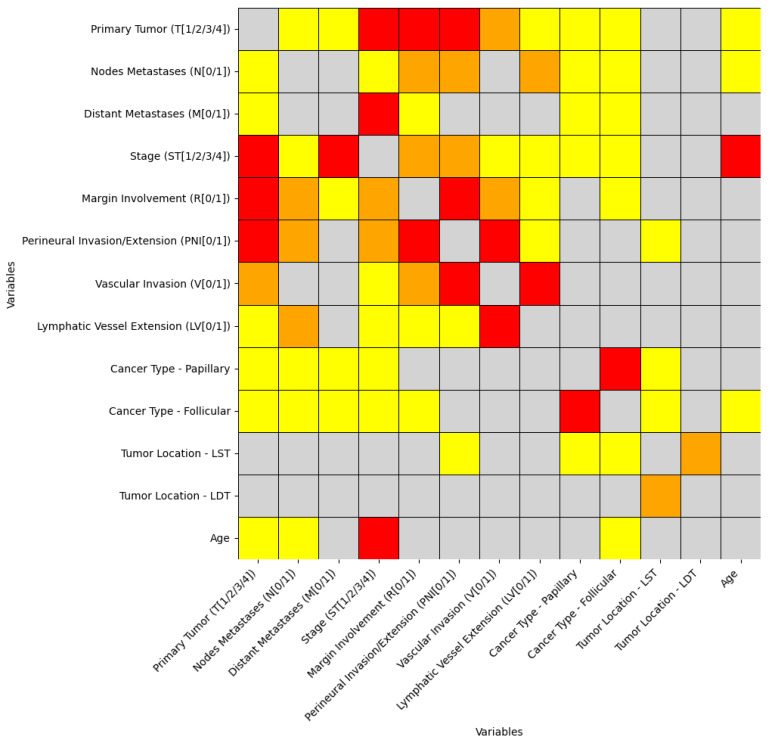
Heatmap of Significant Correlations and Their Effect Sizes. The heatmap includes only variables that show at least one statistically significant correlation with a moderate or large effect size. For these variables, all pairwise correlations are displayed, including insignificant correlations and significant correlations with small effect sizes. Insignificant correlations are shown in grey, small effect sizes in yellow, moderate effect sizes in orange, and large effect sizes in red. Effect sizes (small, moderate, large) were classified using established thresholds specific to each statistical test and corresponding effect size measure (e.g., correlation coefficients, rank-biserial correlation, and dimension-dependent thresholds for Cramér’s V; see Methods for details). All *p*-values were adjusted for multiple testing using the false discovery rate (FDR) correction. (LST-left thyroid lobe, LDT-right thyroid lobe).

The variables for which at least one moderate or large effect size was observed are summarized in Figure 1. As illustrated, only a limited subset of the statistically significant correlations showed moderate or large effect sizes, despite the large number of FDR-adjusted significant results.

### 3.1. Correlations with Moderate and Large Effect Sizes

When stratified by variable type, quantitative–categorical correlations yielded a single association with a large effect size. Categorical–categorical correlations accounted for the majority of non-negligible effects, with eight moderate and eight large effect sizes observed. In contrast, no quantitative–quantitative correlations exhibited moderate or large effect sizes.

**Figure 2:**
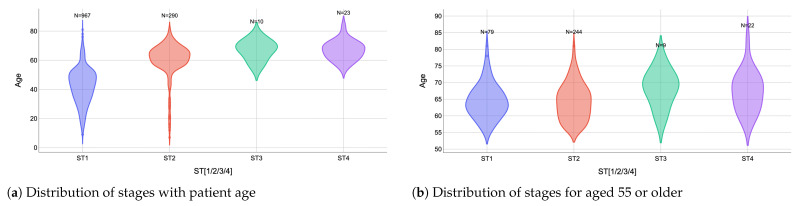
Significant correlations between categorical and numerical variables.

Starting with the correlations between quantitative and categorical variables, the results are summarized in Figure 2. A statistically significant positive association was observed between patient age and disease stage. Specifically, increasing age is associated with higher assigned stages at diagnosis. This relationship is consistent with the staging criteria used in clinical practice, in which patient age is an explicit component of the staging system. Notably, patients younger than 55 years were limited to a maximum assignable stage of ST2, whereas stages ST3 and ST4 were only attainable for patients aged 55 years or older. This constraint is clearly reflected in Figure 2a, where higher-stage observations are exclusively concentrated in the older age group. The magnitude of this association was substantial, as indicated by the large effect size, suggesting that age is a dominant factor in stage determination within the analyzed cohort rather than a marginal or secondary contributor.

When restricting the analysis to patients aged 55 years and older, the association between age and disease stage appears noticeably attenuated. As shown in Figure 2b, the median ages across stages are relatively close (ST1: 64 years; ST2: 64 years; ST3: 69 years; ST4: 67.5 years), with substantial overlap in the age distributions. From a clinical standpoint, this finding is expected and reinforces the interpretation that age functions primarily as a threshold variable in the staging system rather than as a continuous determinant of disease severity once the threshold is crossed. Once the age cutoff of 55 years is reached, further increases in age do not independently increase the assigned stage. Instead, staging within this older subgroup is predominantly governed by tumor-specific and disease-related factors. The modest differences in median age observed between stages are unlikely to be clinically meaningful and may reflect sampling variability rather than a biologically relevant gradient.

**Figure 3:**
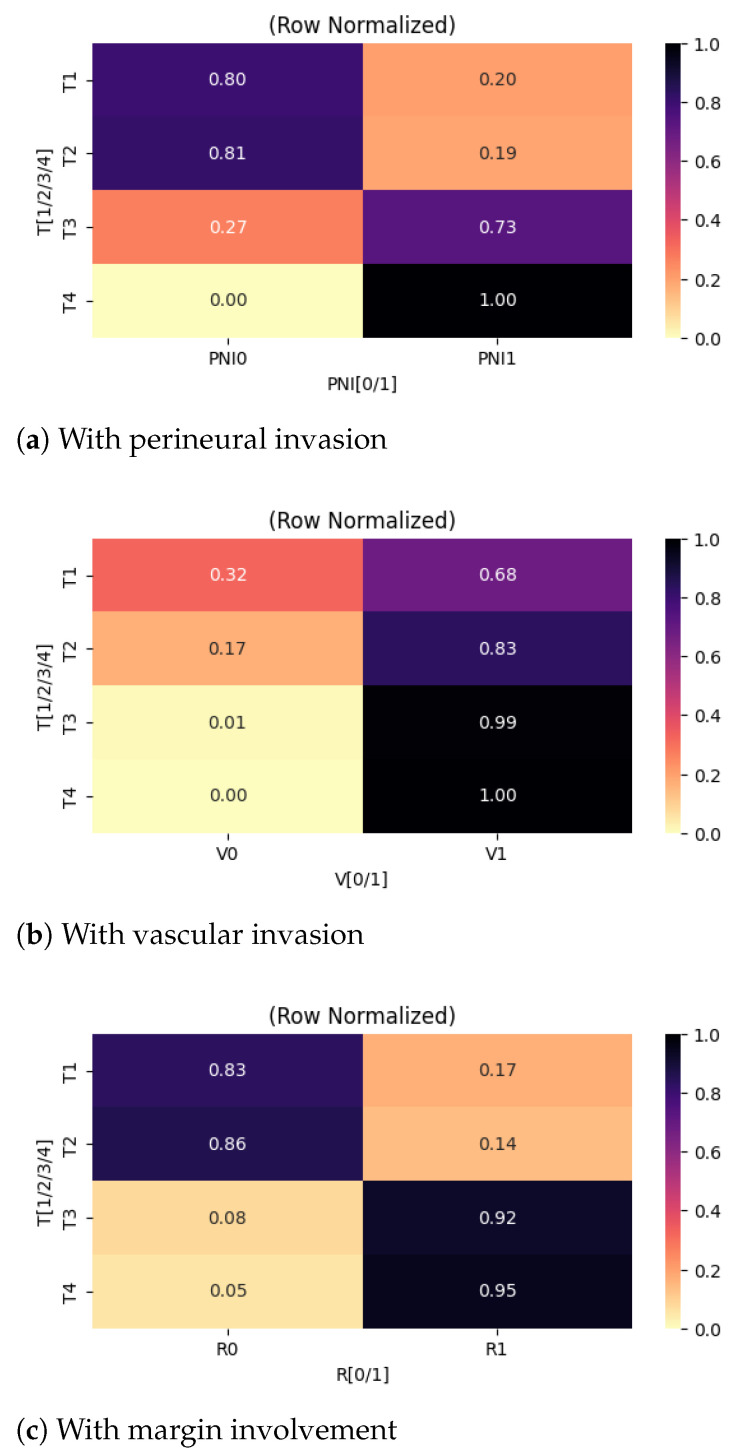
Heatmaps centered on primary tumor categories.

Further on, we examined associations between categorical variables, with an initial focus on correlations involving the primary tumor (T) category. The relationships are summarized using heatmaps presented in Figure 3, enabling a comparative visualization of directional trends across tumor stages.

A strong association was identified between primary tumor category and the presence of perineural invasion. As shown in Figure 3a, increasing tumor stage was associated with a progressively higher prevalence of perineural invasion. From a clinical perspective, this finding is consistent with established pathological mechanisms, as larger or more locally advanced thyroid tumors are more likely to exhibit infiltrative growth patterns and aggressive behavior. Perineural invasion is widely recognized as a marker of adverse prognosis and local aggressiveness, and its correlation with advanced T categories supports its role as a feature of biologically aggressive disease rather than an isolated pathological finding.

Similarly, a strong correlation was identified between tumor category and completeness of surgical resection (Figure 3c). Patients with larger primary tumors (T3 and T4) exhibited higher rates of incomplete resection, with 92% and 95% of cases, respectively, failing to achieve complete tumor removal. From a surgical standpoint, this observation is expected, as advanced tumors are more likely to invade adjacent structures such as the trachea, esophagus, recurrent laryngeal nerve, or major vascular structures, thereby limiting the feasibility of achieving clear surgical margins without unacceptable morbidity.

A moderate association was observed between primary tumor category and vascular invasion (Figure 3b). While the baseline prevalence of vascular invasion was already substantial in early-stage tumors (approximately 68% in T1 tumors), the likelihood increased further with advancing tumor stage. This trend suggests that although vascular invasion can occur early in the disease course, larger tumors are more likely to undergo intravascular spread.

**Figure 4:**
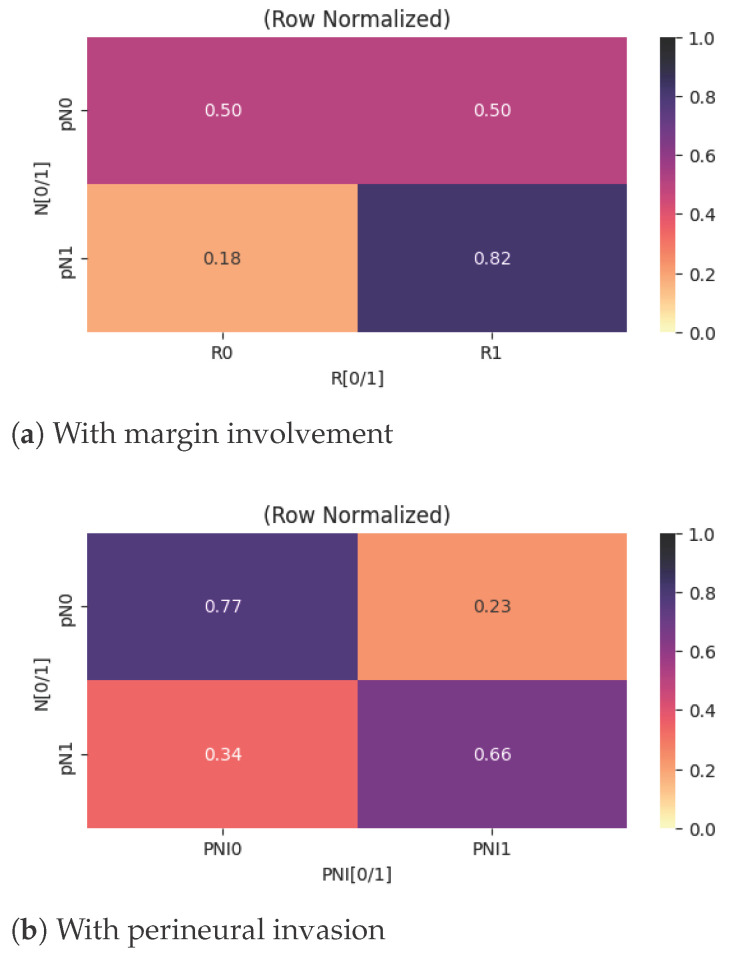
Heatmaps centered on metastases in the regional lymph nodes.

Another evaluated aspect was the presence of metastases in the regional lymph nodes and their associations with other clinicopathological variables. As illustrated in Figure 4a, lymph node metastasis is moderately associated with surgical outcome. In patients without evidence of regional lymph node metastasis, the probability of achieving a complete resection (R0) is approximately 50%. In contrast, among patients with confirmed regional lymph node metastasis, the likelihood of complete resection decreases significantly, to approximately 18%. This finding is consistent with the concept that nodal dissemination reflects more advanced local disease, which may complicate surgical margins and limit the feasibility of achieving complete tumor excision.

With respect to perineural invasion, Figure 4b highlights a moderate, positive correlation with regional lymph node metastasis. Specifically, the probability of identifying perineural invasion increases by approximately 40% in patients who also exhibit metastases in the regional lymph nodes. This association suggests a more aggressive tumor phenotype, as both perineural spread and nodal involvement are commonly regarded as markers of invasive behavior and adverse pathological features in thyroid carcinoma.

**Figure 5:**
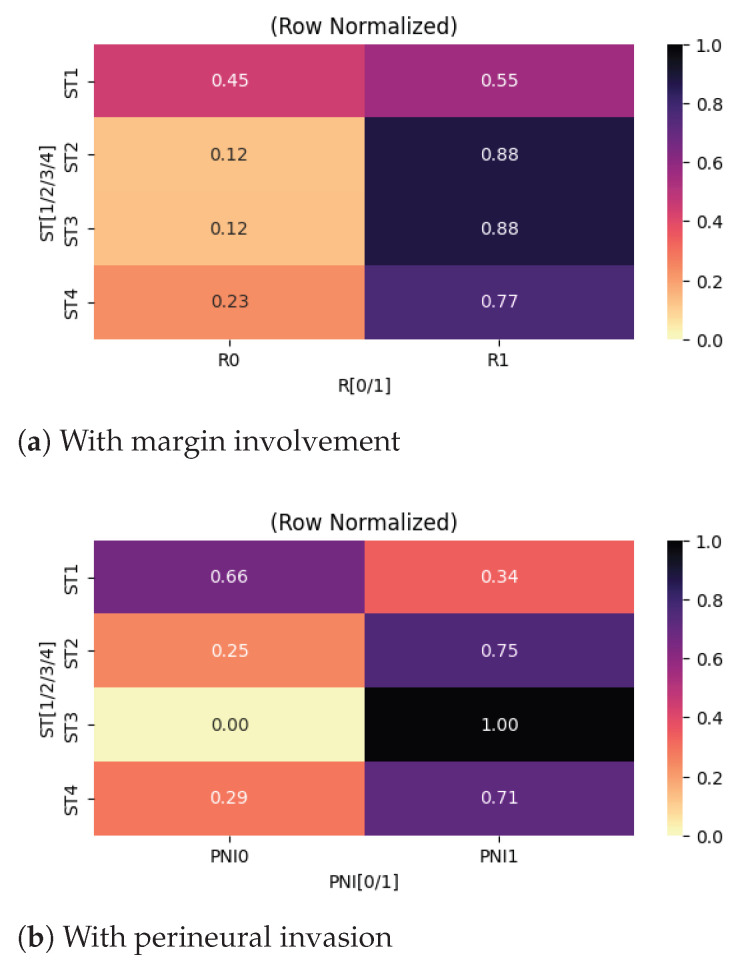
Heatmaps centered on the tumor stage.

The analysis also includes associations between tumor stage and variables with moderate to large effect sizes, as summarized in Figure 5.

The observed moderate correlation between tumor stage and completeness of resection should be interpreted with caution, as the relatively small number of patients at the more advanced stages (ST3 and ST4) may introduce variability in the estimated effect sizes. Nevertheless, as illustrated in Figure 5a, there is a clear and expected trend toward higher rates of complete resections in patients with early-stage disease. From a surgical and oncological perspective, this finding is consistent with the fact that localized tumors without extensive extrathyroidal extension or invasion of adjacent structures are technically more amenable to complete resection with negative margins.

A similar stage-dependent pattern is observed for perineural invasion, as shown in Figure 5b. Lower-stage tumors have a lower frequency of perineural spread, consistent with the biological behavior of less aggressive disease. However, this observation must again be contextualized by the limited number of cases exhibiting perineural invasion in the higher-stage groups. As such, the findings should be interpreted as supportive rather than definitive evidence of a stage-dependent increase in perineural invasion.

**Figure 6:**
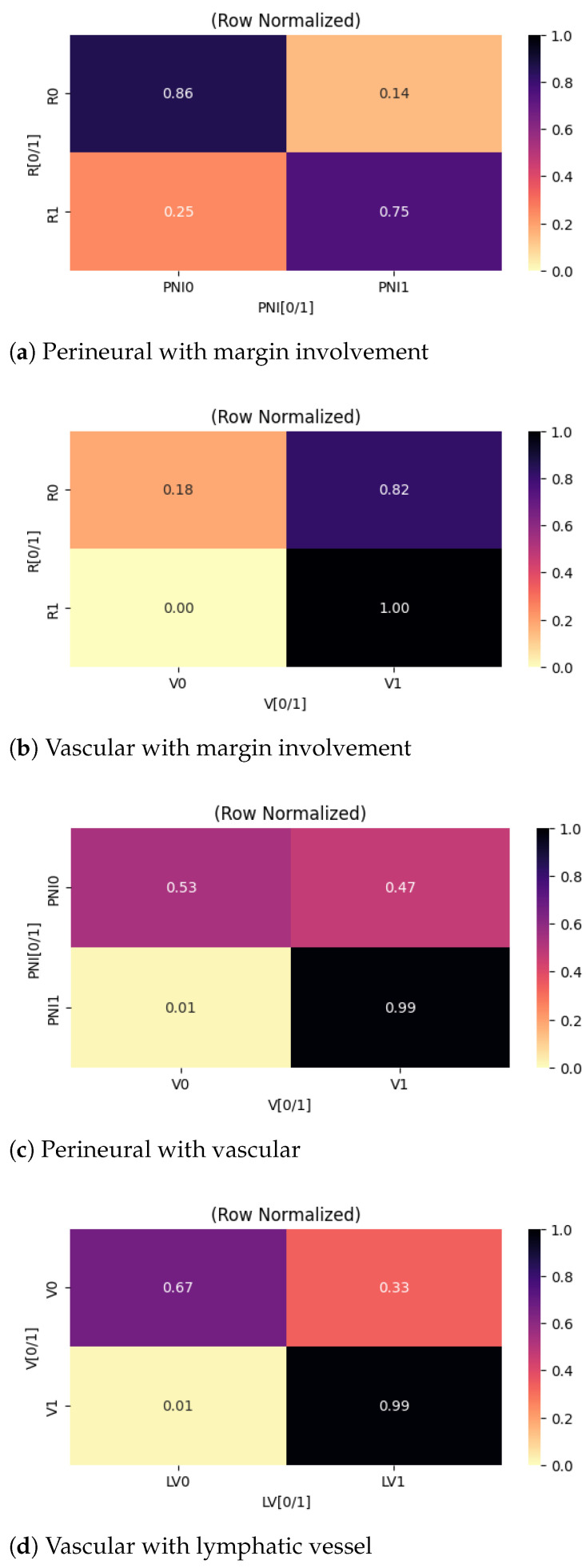
Heatmaps centered on invasions.

Figure 6 further analyzes the associations centered on histopathological indicators of tumor aggressiveness, specifically vascular and perineural invasion. As shown in Figure 6a, the presence of perineural invasion is associated with a higher likelihood of incomplete surgical resection. From a clinical standpoint, perineural spread reflects infiltrative tumor progression along neural sheaths, which may limit the ability to achieve clear surgical margins without unacceptable morbidity. In our cohort, patients with documented perineural invasion were disproportionately represented among cases with incomplete resection, suggesting that this pathological feature may serve as a surrogate marker for technically challenging or biologically aggressive disease.

A similar association is observed for vascular invasion, as illustrated in Figure 6b. Patients with vascular invasion were more likely to undergo incomplete resection. However, the interpretation of this relationship requires additional nuance. In the present population, vascular invasion was highly prevalent, occurring in approximately 82% of patients who achieved complete resection and in 100% of patients with incomplete resection. The high prevalence limits the discriminatory power of vascular invasion as an isolated predictor of resection completeness within this dataset. In contrast, perineural invasion had greater stratification between resection outcomes. Notably, 86% of patients who achieved complete resection lacked evidence of perineural invasion, underscoring its potential role as a more specific indicator of unfavorable local disease characteristics.

We also identified a strong interrelationship between vascular and perineural invasion, as depicted in Figure 6c. Among patients without perineural invasion, the probability of concomitant vascular invasion was approximately 50%. In contrast, vascular invasion was present in nearly all patients with perineural invasion.

Figure 6d denotes a strong association between vascular invasion and lymphatic vessel involvement. Among patients without vascular invasion, lymphatic involvement was observed in approximately one-third of cases. Conversely, lymphatic involvement was nearly universal (99%) for patients with vascular invasion. This finding supports the concept of coordinated vascular and lymphatic dissemination pathways in thyroid carcinoma and reinforces the role of vascular invasion as a marker of systemic metastatic potential rather than purely local aggressiveness.

Collectively, the presented correlations highlight the interconnected nature of invasive histopathological features in thyroid cancer and their cumulative association with suboptimal surgical outcomes.

**Figure 7:**
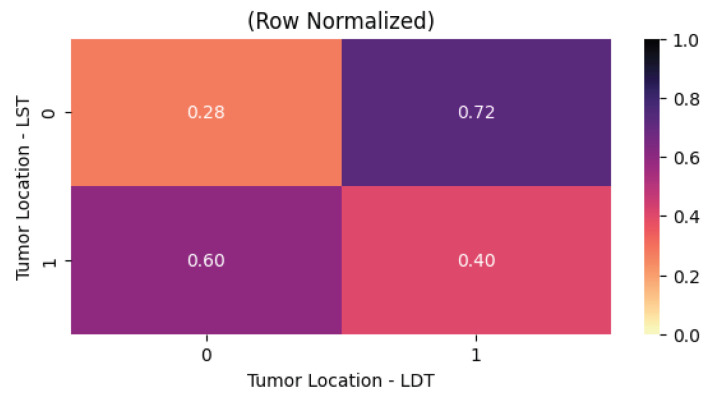
Between the left and right thyroid lobes. (LST-left thyroid lobe, LDT-right thyroid Lobe).

The final statistically significant association with a moderate or strong effect size identified within this data category is summarized in Figure 7. From a clinical and pathological standpoint, this correlation is largely intuitive once the underlying biological and diagnostic constraints are considered. Regarding tumor location, all four anatomical sites in the dataset were evaluated. However, only one meaningful association emerged, namely the correlation between tumors located in the left and right thyroid lobes. This finding is consistent with expected anatomical and probabilistic considerations. In patients diagnosed with thyroid malignancy, tumor presence is far more likely to be confined to a single lobe or a specific subregion of a lobe than to involve multiple distinct anatomical regions simultaneously. Consequently, mutual exclusivity between these two locations produces a detectable negative correlation. This observation reflects not only the typical growth patterns of differentiated thyroid carcinomas but also that multifocal or bilobar disease is more advanced or less common in the studied population.

Lastly, a significant correlation was observed between “Cancer Type—Papillary” and “Cancer Type—Follicular”. This reflects the mutually exclusive nature of these diagnostic categories in the dataset. Because patients are typically classified into a single primary cancer type, the presence of one category inherently implies the absence of the other, producing a strong statistical association that does not represent a clinically meaningful relationship.

### 3.2. Correlations with Small Effect Sizes

The statistically significant associations with small effect sizes emphasize patterns that may be informative despite their limited discriminative power.

The association of age with tumor status (T) and nodal involvement (N) suggests subtle age-dependent differences in disease presentation, a relationship that is often not highlighted, given that age is more commonly viewed as a prognostic rather than morphologic factor.

Among the thyroglobulin-related findings, the association with the sex assigned at birth is comparatively unexpected. While correlations between Tg and tumor- or disease-related variables are biologically plausible given Tg’s role as a marker of tumor burden, sex is not intrinsically linked to Tg production or release. The presence of a statistically significant, albeit low-effect, association therefore suggests subtle sex-related differences in Tg levels or their interpretation.

More unexpected is the association between anti-thyroglobulin antibodies and distant metastasis (M), in contrast to their associations with tumor stage (ST), resection status (R), lymphovascular invasion (LV), or sex. Given that Anti-Tg is generally regarded as an immunological or analytical modifier rather than a tumor-related marker, its correlation with metastatic status, even if weak, suggests underlying interactions between the immune response and advanced disease.

Regarding associations between categorical variables, the associations between sex and multiple indicators of disease extent, including primary tumor status (T), nodal involvement (N), and distant metastasis (M), are noteworthy. Although each association is weak, their consistency across different dimensions of tumor spread suggests that sex may be linked to modest differences in disease presentation or detection. As sex is not intrinsically related to tumor morphology, these correlations merit acknowledgment without implying a direct biological mechanism.

Finally, the associations involving tumors located in the isthmus and both tumor and nodal status warrant brief mention. Given the anatomical characteristics of isthmic tumors, the presence of significant, albeit weak, correlations with disease extent suggests that tumor location may subtly influence presentation.

Collectively, these findings emphasize that statistically significant low-effect associations can expose nuanced patterns that complement, rather than redefine, established clinicopathological frameworks.

### 3.3. Subtype Analysis

We additionally investigated potential correlations between thyroid cancer subtypes and all other available variables. Following this analysis, several variables achieved statistically significant associations with cancer subtype after correction for multiple testing. Moderate associations were observed for V, LV, N, and ST variables, with Cramér’s V values ranging from 0.1869 to 0.4875. Stronger associations were observed for T, R, and PNI variables, with Cramér’s V values ranging from 0.4216 to 0.5653, indicating a greater dependence on cancer subtype. The corresponding heatmaps are provided in the Supplementary Materials File S2 (i.e., subtype_heatmaps.docx) and may serve as a reference for domain specialists.

Furthermore, subtype-specific estimates were computed for all variables included in the analysis. Owing to space constraints, Table 3 reports estimates for the three most prevalent subtypes in the study cohort—papillary microcarcinoma, diffuse sclerosing variant of papillary thyroid carcinoma (PTC), and classical variant of papillary thyroid carcinoma. Estimates for the remaining subtypes, including the follicular variant of PTC, mixed PTC/FTC variant, tall cell variant of PTC, encapsulated variant of PTC, oncocytic variant of PTC, minimally invasive FTC, solid–trabecular variant of PTC, follicular thyroid carcinoma (FTC), and rare PTC/FTC variants (n < 10), are provided in the Supplementary Material File S1 (i.e., subtype_estimates.xlsx).

As a final step of the exploratory analysis, two regression models were fitted to predict lymph node metastasis (N) and margin involvement (R) using the predictors described in the Section 2. Each model was applied separately to patient groups defined by cancer subtype. The analyses focused on the three most prevalent cancer subtypes in the cohort, as presented in Table 3.

In papillary microcarcinoma, the regression model for lymph node metastasis was applied to 203 cases (CS pseudo-R^2^ = 0.15). Male sex was independently associated with lymph node metastasis (*p* = 0.004), indicating a higher nodal risk than in female patients, while tumor size category showed a borderline association with nodal involvement (*p* = 0.081), suggesting a size-related trend even within microcarcinomas. Age and isthmus location were not significantly associated with nodal status in the proposed model. Lymphovascular invasion displayed a very large coefficient, with an inflated standard error and a large *p*-value, indicating quasi-complete separation. Cross-tabulation illustrated only 30 cases without such invasion, all without lymph node metastasis, indicating a strong but near-deterministic relationship with nodal metastasis in this subtype. The results suggest that lymph node metastasis in papillary microcarcinoma may be associated with patient sex and invasive growth characteristics, with tumor size showing a potential but non-significant trend.

The regression model for margin involvement in papillary microcarcinoma was applied to 139 cases (CS pseudo-R^2^ = 0.51). Tumor size category emerged as an independent predictor of margin positivity, with larger tumors showing a markedly increased likelihood of R1 resection (*p* < 0.001). Nodal involvement was also independently associated with margin positivity (*p* = 0.011), indicating that more extensive disease correlated with incomplete clearance. Sex, age, isthmus location, and lymphovascular invasion were not significantly associated with margin status in the proposed model. It is worth noting that only 1 case with lymphovascular invasion showed margin involvement in this sample. Taken together, the findings indicate that margin involvement in papillary microcarcinoma may be more closely related to measures of tumor burden and disease extent than to demographic factors or anatomic location, acknowledging limited information on lymphovascular invasion in this cohort.

In the diffuse sclerosing variant of PTC, the regression model for lymph node metastasis was applied to 222 cases (CS pseudo-R^2^ = 0.06). Male sex was independently associated with lymph node metastasis (*p* = 0.018), and increasing age was also significantly associated with nodal positivity (*p* = 0.041), indicating that host-related factors may contribute to nodal risk in this phenotype. Tumor size category and isthmus location were not significantly associated with nodal status after adjustment. Lymphovascular invasion was ubiquitous for this subtype and therefore not discriminative. Influence diagnostics identified several high-leverage observations, all of which corresponded to node-negative cases. These results suggest that lymph node metastasis in the diffuse sclerosing variant of PTC may arise against a background of ubiquitous lymphovascular invasion, with limited modulation by local tumor features and modest associations with patient-related factors such as sex and age.

The regression model for margin involvement in the diffuse sclerosing variant of PTC was applied to 175 cases (CS pseudo-R^2^ = 0.12). Tumor size category was the only variable independently associated with margin positivity (*p* < 0.001), with higher tumor categories showing an increased likelihood of R1 resection. There were no cases without lymphovascular invasion in this sample. Nodal status, age, sex, and isthmus location were not significantly associated with margin involvement after adjustment, suggesting that margin positivity in this phenotype may be driven primarily by local tumor burden rather than overall disease extent or patient-related factors.

In the classical variant of papillary thyroid carcinoma, the regression model for lymph node metastasis was applied to 138 cases (CS pseudo-R^2^ = 0.10). Increasing age had a borderline association with nodal metastasis (*p* = 0.053), suggesting a trend toward higher nodal risk in older patients, while sex, tumor size category, and isthmus location were not independently associated with nodal status after adjustment. Lymphovascular invasion displayed a very large coefficient accompanied by an inflated standard error and a large *p*-value, reflecting quasi-complete separation; cross-tabulation confirmed only 2 cases without such invasion, all without lymph node metastasis, indicating a strong but near-deterministic relationship with nodal metastasis in this subtype. These results indicate considerable residual variability in nodal status in classic PTC that is not captured by the modeled clinicopathologic features, with a borderline association with increasing age, whereas lymphovascular invasion shows a strong but statistically unstable association.

The regression model for margin involvement in classic PTC was applied to 92 cases (CS pseudo-R^2^ = 0.32). Tumor size category was associated with margin positivity (*p* < 0.001), with higher tumor categories associated with a likelihood of incomplete resection. Lymphovascular invasion was also independently associated with margin involvement (*p* = 0.003), indicating that infiltrative growth patterns may contribute to surgical margin positivity in this subtype. However, it is worth noting that only 1 case with lymphovascular invasion showed margin involvement in this sample. In contrast, nodal status, age, sex, and isthmus location were not significantly associated with margin status after adjustment. These results suggest that in classic PTC, margin involvement may be primarily driven by tumor size and invasive growth characteristics, rather than by patient-related factors or tumor location.

Overall, this exploratory analysis highlights potential distinct and subtype-specific patterns in the factors associated with lymph node metastasis and margin involvement. Lymphovascular invasion did not emerge as a reliable discriminator across models; it was nearly ubiquitous in analyses of nodal involvement and, conversely, rare in analyses of margin involvement.

Demographic factors may be associated with an increased risk of nodal metastasis, with subtype-specific patterns. In papillary microcarcinoma, male sex may be associated with a higher risk of nodal involvement. In the diffuse sclerosing variant, both male sex and increasing age may be associated with a higher risk of nodal involvement. In the classical variant of papillary thyroid carcinoma, increasing age showed a borderline association with a higher risk of nodal involvement. However, these subtype-specific associations may reflect model misspecification, and further analyses are warranted to confirm their robustness.

Tumor burden, as reflected by tumor size category, emerged as a consistent predictor of margin involvement across subtypes, underscoring the potential role of local disease extent in surgical clearance. Nodal involvement additionally showed a signal for margin positivity in papillary microcarcinoma, suggesting that regional disease spread may further complicate complete resection in this subtype.

## 4. Discussion

### 4.1. Comparison to Existing Literature

Across the current analysis, greater tumor burden was consistently associated with invasive pathological features and with markers of more complex surgical management, in line with findings from the current thyroid cancer literature. In bivariate analyses, a higher primary tumor category (T) was associated with increased frequencies of nodal metastases (N), perineural invasion (PNI), and vascular invasion (V), as well as higher rates of margin involvement (R). The associations are consistent with established principles in surgical oncology, whereby locally advanced tumors are more likely to exhibit infiltrative growth and are more difficult to excise completely at the microscopic level [33]. Together, the findings support the view that these features tend to occur within a shared aggressive tumor profile.

Within the analyzed cohort, perineural invasion was most frequently observed in tumors with advanced T category, nodal metastasis, and margin involvement, and it showed a moderate association with lymph node involvement. Previous studies describe perineural invasion in papillary thyroid carcinoma as an uncommon feature that is often accompanied by other high-risk pathological characteristics. For example, matched cohort analyses have reported associations between perineural invasion and lymphovascular invasion, microscopic extrathyroidal extension, increased nodal burden, and higher rates of extranodal extension in node-positive disease [34]. However, whether perineural invasion independently influences prognosis remains uncertain. Propensity-score–matched analyses suggest that its apparent association with recurrence and lateral neck metastasis may be explained by coexisting aggressive features, such as gross extension, vascular invasion, and nodal disease, rather than by a direct effect of perineural invasion itself [35]. In this context, the bivariate results in our data are consistent with perineural invasion being part of a broader invasive growth pattern, highlighting the importance of adequate confounder adjustment in prognostic analyses.

Furthermore, the weak but statistically significant associations observed for thyroglobulin and anti-thyroglobulin antibodies should be interpreted with caution. It has been acknowledged that anti-thyroglobulin antibodies, while immunological markers and potential indicators of recurrence risk [36], also function as analytical interferents that complicate the interpretation of thyroglobulin measurements [37]. Therefore, observed correlations between anti-thyroglobulin antibodies and disease extent may reflect a combination of biological factors, such as tumor–immune interactions, and measurement-related limitations rather than a direct causal relationship. Prior research has reported associations between preoperative thyroid function markers, elevated thyroid-stimulating hormone, thyroglobulin antibodies, and invasive features in univariate analyses. However, these associations often attenuate or lose significance after multivariable adjustment and show inconsistent relationships with tumor stage [38,39]. The pattern is consistent with the modest effect sizes observed in our cohort and suggests that these biochemical markers are more likely to indicate underlying immune or physiological states than to directly trigger tumor invasiveness.

Exploratory subtype-stratified models identified tumor size category as a predictor of margin involvement (R) across subtypes. This pattern may suggest that increasing disease extent complicates surgical resection and raises the likelihood of residual disease. At the same time, the clinical significance of microscopic margin involvement in well-differentiated thyroid cancer remains debated [40]. A 2021 review found no consistent association between microscopic positive margins and local recurrence, highlighting variability in margin definitions and frequent overlap with extrathyroidal extension [40]. More recent studies similarly noted that most positive margins in thyroid cancer are microscopic and that their prognostic impact is uncertain, particularly in the context of effective adjuvant therapy and close surveillance [41,42].

Large meta-analyses indicated that tumor size, capsular or extrathyroidal invasion, tumor location, age, and sex are the most consistent predictors of lymph node metastasis in papillary thyroid carcinoma [43]. Overall, the bivariate analyses broadly supported this perspective, though tumor location and sex did not show moderate or large effect sizes. Furthermore, subtype-specific analyses suggest that the influence of these factors may vary across subtypes.

Furthermore, in papillary microcarcinoma (PTMC), male sex was independently associated with lymph node metastasis, with tumor size showing a borderline association. These exploratory findings align with recent evidence suggesting that a subset of small tumors can exhibit aggressive behavior and that sex and size thresholds may help identify higher-risk PTMC. For instance, a 2025 clinicopathologic study of tumors ≤1.0 cm identified male sex and tumor size >5 mm as factors associated with aggressive features [44]. Other recent PTMC studies have instead identified younger age as a stronger predictor of nodal involvement after adjustment [45,46].

In the classical variant of papillary thyroid carcinoma, lymph node metastasis showed only weak or borderline associations with age. Lymphovascular invasion appeared associated with nodal disease but showed statistical instability due to quasi-complete separation, with few lymphovascular invasion–negative cases among node-positive tumors. This denotes a common modeling challenge in thyroid cancer datasets, where strong clinical associations may yield unstable estimates when predictor-outcome combinations are rare [47].

Since the presence of risk factors indicates a possible increase in biological aggressiveness, adequate postoperative therapy and close follow-up are essential. The present analysis regarding the diffuse sclerosing variant (DSV) shows that lymph node metastasis was highly prevalent, and the regression model explained little variance. This pattern is consistent with DSV being an intrinsically lymphotropic subtype, in which nodal involvement is common regardless of tumor size or location [48]. Recent studies describe frequent nodal metastasis and higher recurrence rates in DSV, particularly among younger patients, while disease-specific mortality remains low [49,50]. The relatively high number of DSV cases in this cohort compared with typical prevalence estimates (approximately 0.8–5.3% of PTC) suggests possible referral bias or differences in histopathological classification, which should be considered when comparing absolute rates across studies [49].

Finally, isthmus involvement did not independently predict lymph node metastasis or microscopic margin involvement in the multivariable models. This contrasts with several recent studies reporting distinct nodal spread patterns and an increased risk of central lymph node metastasis in isolated isthmic papillary thyroid carcinoma [51,52]. The discrepancy may reflect differences between tumors predominantly confined to the isthmus versus those with any isthmus involvement, the limited predictor set used to maintain model identifiability in subtype analyses, or unmodeled treatment-related factors such as the extent of surgery and lymph node dissection.

Beyond these findings, the existing literature underscores the particular relevance of differentiated thyroid cancer in the Romanian context. Notably, DTC has a high survival rate in Romania, with some pediatric cases reaching up to 100%. However, the increasing number of cases poses a significant financial burden. A study conducted in Mureș County from 1990 to 2009 showed a clear rise in thyroid cancer incidence, primarily due to small papillary cancers, while undifferentiated cancers have seen a decline. The result emphasizes the urgent need for improved morphological criteria to explain the rising rates of the follicular variant of papillary carcinoma. Additionally, the incidence of anaplastic carcinoma was notably high at 20% in the first decade but decreased sharply to 3.46% after 2000 (*p* = 0.00002) [53].

A comprehensive six-year retrospective study (2017–2022) at the Emergency County Hospital in Târgu-Mureș indicated a significant increase in malignant tumor incidence during the COVID-19 pandemic, rising from 26.3% to 35.4% (*p* = 0.002), with papillary thyroid carcinoma being the most prevalent type [54].

To address these challenges, improved management strategies are required, particularly those grounded in refined risk stratification. Enhanced stratification has the potential to reduce unnecessary interventions and generate economic benefits by optimizing clinical decision-making. Such advances increasingly rely on comprehensive data analysis and modeling, which can provide insights beyond those obtained through traditional statistical summaries.

Within the Romanian context, modeling efforts have already demonstrated their value. Spatial analyses based on geographic information systems (GIS) have revealed significant geographic clustering of thyroid cancer cases, highlighting regions that may be relevant for etiological investigation and targeted surveillance [55]. Notably, despite a slight decline in national pediatric cancer prevalence—from 3.57‰in 2008 to 3.44‰in 2017—persistent high-risk clusters have been reported in Central and South-Eastern Romania, particularly in historically industrialized counties [56].

Building on this line of research, data-driven statistical modeling offers a complementary perspective by enabling the exploration of correlations and potential causal pathways at the population level. In this context, correlation-based modeling emerges as a valuable tool for characterizing risk factors, with potential implications for prognostic assessment, resource allocation, and public health decision-making in differentiated thyroid cancer.

### 4.2. Principal Challenges and Limitations

Several limitations should be considered when interpreting the findings and assessing their generalizability. First, the study’s retrospective, single-center design and treatment-based selection may influence the patient population. All included patients underwent thyroid surgery followed by radioiodine therapy, which likely enriches the cohort for individuals managed with more intensive treatment strategies than those currently eligible for active surveillance or lobectomy alone, particularly among patients with small papillary tumors. This pathway-based selection may alter the distribution of risk factors and modify observed associations relative to population-based or surveillance-focused cohorts.

Second, the analyzed outcomes reflect cross-sectional clinicopathologic associations rather than long-term prognosis. The exploratory regression models examine predictors of lymph node metastasis and margin involvement but do not address recurrence-free survival, disease-specific survival, or response to therapy. Consequently, the study cannot determine whether features such as perineural invasion or margin involvement provide independent prognostic information beyond their correlation with other high-risk characteristics.

Third, heterogeneity in pathological reporting may affect interpretation. A recent study [57] has shown that definitions of vascular invasion in thyroid carcinoma are inconsistently applied, and that distinguishing lymphatic from venous invasion is often impractical in routine histopathology. The strong coupling observed between vascular and lymphovascular invasion in this cohort may therefore reflect true biological co-occurrence, differences in reporting practices, or the use of “vascular invasion” as a broad umbrella term rather than a strictly defined entity.

Fourth, despite the use of false discovery rate correction and effect size reporting, bivariate associations remain susceptible to confounding and collider bias. For example, treatment intensity may influence both lymph node yield and margin status, creating apparent associations that do not reflect direct biological relationships. The large number of statistically significant but small-effect findings likely includes associations that are detectable due to sample size rather than for their clear clinical relevance.

Fifth, subtype-stratified analyses were impacted by limited sample sizes and quasi-complete separation, particularly for lymphovascular invasion. This led to inflated coefficient estimates and unstable *p*-values in some models, despite signals in cross-tabulated data. Such behavior is a known limitation of standard maximum-likelihood logistic regression in sparse or imbalanced strata and restricts inference about independent effects within specific subtypes.

Sixth, the multivariable models were deliberately limited to preserve identifiability, but this restriction leaves room for unmeasured confounding. Several factors known to influence nodal metastasis and margin status—such as multifocality, extrathyroidal extension, number of lymph nodes removed, extranodal extension, tall-cell histologic features, and molecular alterations (e.g., BRAF, TERT, RET) were not included. Their omission may limit the mechanistic interpretation of the observed associations.

Finally, issues of generalizability should be acknowledged. The cohort was predominantly composed of papillary thyroid carcinoma cases, reflecting the epidemiological distribution of thyroid cancer in clinical practice. Smaller numbers of follicular thyroid carcinoma and mixed histology tumors were also present, consistent with the diversity observed in routine clinical settings. The analyses were therefore performed on the cohort as a whole; however, due to the predominance of papillary thyroid carcinoma, this subtype contributes more strongly to the overall results. Additionally, the observed prevalence of certain phenotypes, particularly the diffuse sclerosing variant, appears higher than typically reported in large series (approximately 0.8–5.3%) [49]. This may reflect patient selection related to radioiodine therapy patterns, institutional expertise, or differences in histopathological classification. While such discrepancies do not undermine the validity of associations observed within the cohort, they warrant caution when comparing absolute frequencies across different clinical settings.

## 5. Conclusions

In the presented exploratory analysis of a large, contemporary cohort of differentiated thyroid cancer patients undergoing surgical and radioiodine treatment, we combined effect–size–aware bivariate analysis with subtype-specific multivariable modeling to characterize the interrelationships among demographic, clinical, biochemical, and histopathological features.

The present exploratory analysis highlights several findings that may inform future research and clinical interpretation of datasets on differentiated thyroid cancer. The present study illustrates the following main conclusions:

- Interrelated invasive histopathological features. Moderate to large effect size correlations highlight the interdependence among invasive pathological characteristics, particularly vascular invasion, lymphovascular invasion, and perineural invasion. These features also showed associations with nodal metastasis and margin involvement, suggesting that aggressive tumor characteristics tend to occur together rather than in isolation.
- Tumor burden and surgical outcomes. Increasing tumor size category was consistently associated with a higher likelihood of margin involvement across the analyzed subtypes, indicating that greater tumor burden may contribute to more challenging surgical clearance.
- Exploratory subtype-related patterns. Subtype-stratified analyses suggested that some clinicopathological associations, particularly those related to nodal metastasis, may vary across thyroid cancer subtypes. However, these observations should be interpreted cautiously and primarily as hypothesis-generating.
- Descriptive estimates for future research. The study also provides subtype-specific confidence intervals for key clinicopathological and biochemical variables, which may serve as reference values for future studies and comparative analyses.

Future work will focus on strengthening the clinical relevance and generalizability of the findings by moving beyond cross-sectional associations toward outcome-oriented and longitudinal analyses. Incorporating follow-up data would enable evaluation of whether histopathologic and biologic features contribute independent prognostic information when considered alongside established measures of disease extent. At the same time, expanding cohort size, enriching clinical covariates, and adopting modeling strategies designed to handle sparse or imbalanced data would support more stable inference across disease subtypes and reduce sensitivity to correlated high-risk features. External validation through multi-center collaboration would help distinguish institution-specific effects from reproducible patterns and improve confidence in the applicability of the results across settings.

## Figures and Tables

**Table 1:** Categorical variables summary.

Variable	Category	n	Variable	Category	n
Sex	Female	1160	Cancer Type	PTC	1403
	Male	310		FTC	82
				MTC	8
				Mixed *	23
Primary Tumor Stage	T1	565	Tumor Location	LST ^†^	781
	T2	149		LDT ^†^	815
	T3	692		Isthmus	206
	T4	27		Pyramidal Lobe	8
	Missing	37		Mixed *	402
				Missing	133
Nodal Status (N)	N0	445	Distant Metastasis (M)	M0	1355
	N1	490		M1	51
	Missing	535		Missing	64
Clinical Stage (ST)	ST1	967	Margin Involvement (R)	R0	342
	ST2	290		R1 (+R2)	567
	ST3	10		Missing	561
	ST4	23			
	Missing	180			
Vascular Invasion (V)	V0	31	Lymphovascular (LV)	LV0	104
	V1	347		LV1	1053
	Missing	1092		Missing	313
Perineural Invasion (PNI)	PNI0	244			
	PNI1	182			
	Missing	1044			

* For cancer type, subtracting mixed cases is sufficient to recover the total of 1470 cases because overlaps involve only two categories at a time. For tumor location, cases may overlap across 2, 3, or 4 categories, so subtracting mixed cases alone does not recover the total due to recoding. ^†^ LST-left thyroid lobe, LDT-right thyroid lobe.

**Table 2:** Quantitative variables summary.

Variable	Mean	St. Dev.
Age	48.63	14.61
Thyroglobulin (TG)	162.96	1937.36
Anti-Thyroglobulin (Anti-TG)	105.64	489.24
Thyroid Stimulating Hormone (TSH)	132.09	2606.66

**Table 3:** Subtype-specific 95% confidence intervals for clinicopathologic variables. For categorical variables, values represent confidence intervals for proportions (%), while for numerical variables they represent confidence intervals for means. Confidence intervals for proportions were calculated using the Wilson method, and confidence intervals for means using a t-distribution-based method. Similar information for additional subtypes is provided in the Supplementary Material.

Variable	Papillary Microcarcinoma	Diffuse Sclerosing PTC	Classic PTC
Sex (F)	84.56% (80.73–87.74%)	78.20% (73.09–82.57%)	76.58% (70.58–81.67%)
Tumor location (LST)	55.88% (51.03–60.62%)	54.67% (48.91–60.31%)	50.90% (44.37–57.41%)
Tumor location (LDT)	55.15% (50.30–59.90%)	58.82% (53.07–64.35%)	55.41% (48.83–61.80%)
Tumor location (Isthmus)	13.97% (10.94–17.67%)	19.03% (14.92–23.95%)	15.77% (11.56–21.14%)
Tumor location (Pyramidal lobe)	1.23% (0.52–2.84%)	0.69% (0.19–2.49%)	0.00% (0.00–1.70%)
T1	76.68% (74.44–82.37%)	12.46% (9.14–16.76%)	34.23% (28.31–40.70%)
T2	0.00% (0.00–0.93%)	3.81% (2.14–6.69%)	11.71% (8.21–16.61%)
T3	20.59% (16.95–24.78%)	80.28% (75.30–84.45%)	44.59% (38.20–51.17%)
T4	0.00% (0.00–0.93%)	3.11% (1.65–5.81%)	1.35% (0.46–3.90%)
N positive	25.00% (19.91–30.90%)	78.76% (72.97–83.59%)	70.70% (63.16–77.26%)
M positive	0.51% (0.14–1.84%)	3.23% (1.71–6.02%)	5.21% (2.94–9.09%)
Stage I	69.36% (64.73–73.64%)	66.78% (61.16–71.96%)	70.27% (63.95–75.90%)
Stage II	12.99% (10.07–16.60%)	25.26% (20.60–30.57%)	18.47% (13.92–24.09%)
Stage III	0.00% (0.00–0.93%)	1.04% (0.35–3.01%)	0.45% (0.08–2.51%)
Stage IV	0.00% (0.00–0.93%)	1.73% (0.74–3.99%)	0.45% (0.08–2.51%)
Margin involvement (R+)	36.67% (33.82–42.93%)	92.51% (88.34–95.27%)	58.39% (50.02–66.31%)
Perineural invasion (PNI+)	21.21% (15.10–28.85%)	74.63% (63.07–83.51%)	41.57% (31.89–51.95%)
Vascular invasion (V+)	84.44% (71.22–92.25%)	98.77% (93.33–99.78%)	84.21% (69.58–92.56%)
Lymphovascular invasion (LV+)	86.45% (82.34–89.71%)	100.00% (98.63–100.00%)	94.68% (90.49–97.09%)
Age (years)	51.45 (50.22–52.69)	45.27 (43.46–47.09)	44.38 (42.31–46.45)
Thyroglobulin (ng/mL)	17.68 (10.52–24.83)	108.99 (41.33–176.64)	76.50 (24.36–128.64)
Anti-TG (UI/mL)	60.58 (41.89–79.27)	181.99 (84.49–277.51)	140.70 (66.37–215.03)
TSH (μUI/mL)	62.63 (60.33–64.94)	66.37 (62.95–69.79)	67.01 (63.50–70.52)

## Data Availability

The dataset derived from patient records and analyzed during this study is not publicly available due to privacy and ethical considerations, but is available from the corresponding authors upon reasonable request.

## Supplementary Materials

The following supporting information can be downloaded at: https://www.mdpi.com/article/10.3390/cancers18061036/s1, File S1: subtype_estimates.xlsx: An Excel table reporting, for each cancer subtype and variable in the dataset, the percentage or mean (first line), the corresponding 95% confidence interval (second line), and the sample size used for the calculation (third line). File S2: subtype_heatmaps.docx: A document reporting the detailed results of the correlation analysis between thyroid cancer subtypes and the remaining variables in the dataset, including the exact statistical values underlying the subtype-specific heatmap analyses. File S3: heatmaps_unnormalized.docx: A document presenting the unnormalized values for correlations with moderate and large effect sizes identified in the bivariate analysis.

## Abbreviations

The following abbreviations are used in this manuscript:

Anti-TG	Anti-Thyroglobulin Antibodies
ATA	American Thyroid Association
CIPNIE	Romanian National Institute of Endocrinology “C.I. Parhon”
DSV	Diffuse Sclerosing Variant
DTC	Differentiated Thyroid Cancers
EHR	Electronic Health Record
FDR	False Discovery Rate
FTC	Follicular Thyroid Carcinoma
GDPR	General Data Protection Regulation
LV	Lymphovascular Invasion
LST	Left Thyroid Lobe
LDT	Right Thyroid Lobe
M	Distant Metastasis Status
N	Nodal Status
OTC	Oncocytic Thyroid Carcinoma
PNI	Perineural Invasion
PTC	Papillary Thyroid Carcinoma
PTMC	Papillary Microcarcinoma
R	Tumor Margin Involvement
ST	Clinical Stage
T	Primary Tumor Stage
TC	Thyroid Cancer
Tg	Thyroglobulin
TSH	Thyroid-Stimulating Hormone
V	Vascular Invasion
WBS	Whole-Body Scintigraphy
WHO	World Health Organization
