# Supplementary material for: Exploratory Statistical Analyses of Clinical and Biochemical Factors for Differentiated Thyroid Cancer from a Romanian Cohort
Source: Cancers (Basel). 2026 Mar 23;18(6):1036. doi: 10.3390/cancers18061036 (PMC13025962; doi:10.3390/cancers18061036)
Supplement: Supplementary file 1 [file cancers-18-01036-s001.zip › File S2-subtype_heatmaps.pdf]

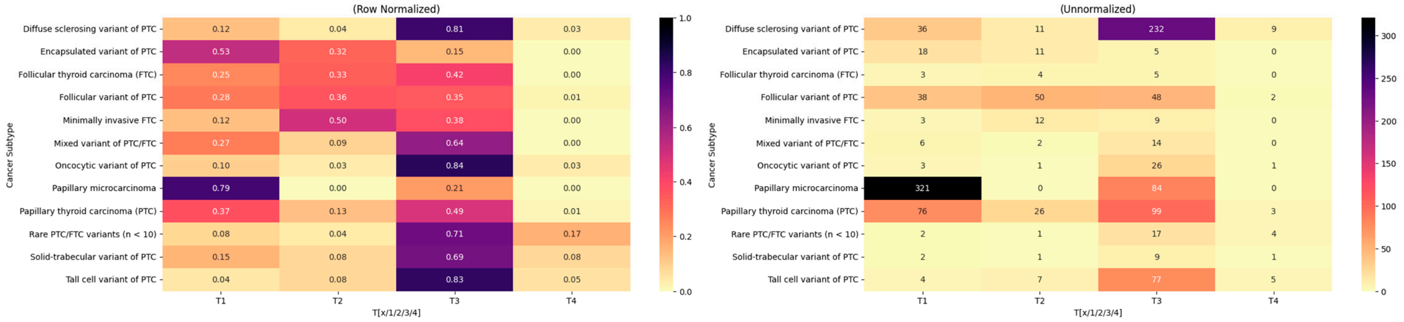

Figure S1. Heatmap Subtype – Primary Tumor

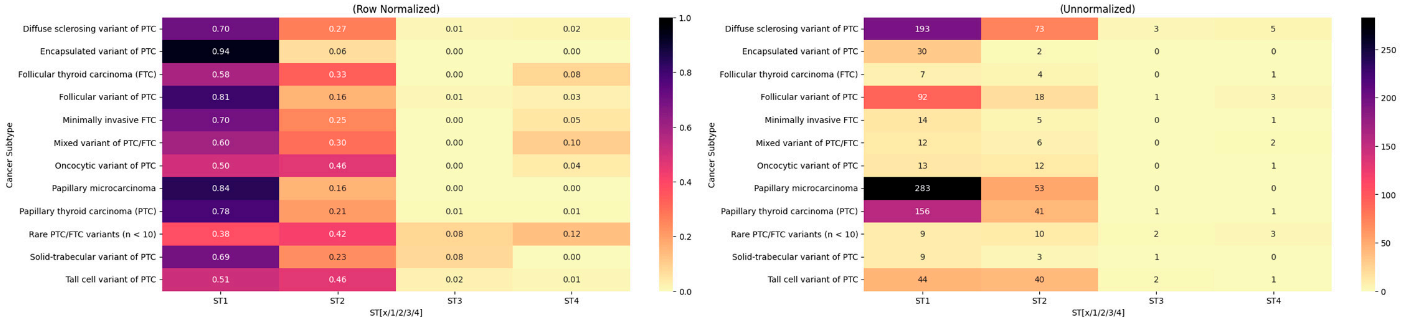

Figure S2. Heatmap Subtype - Stage

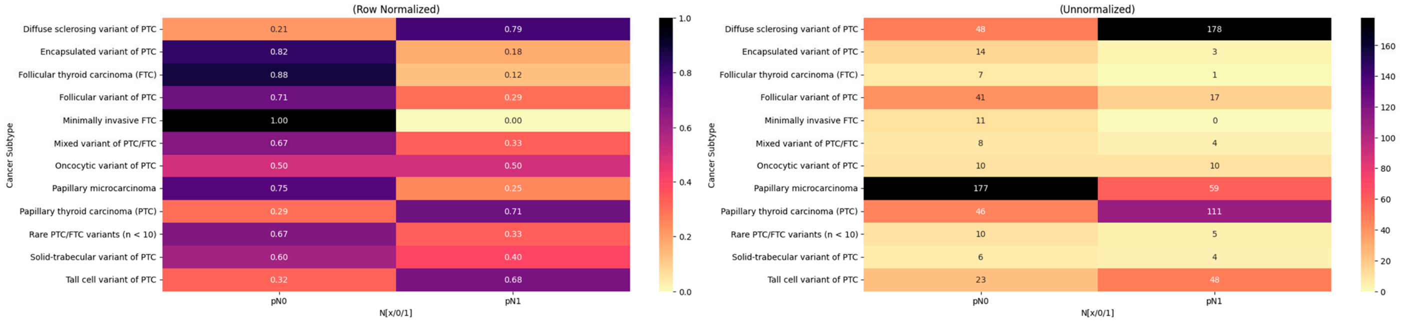

Figure S3. Heatmap Subtype – Nodes Metastases

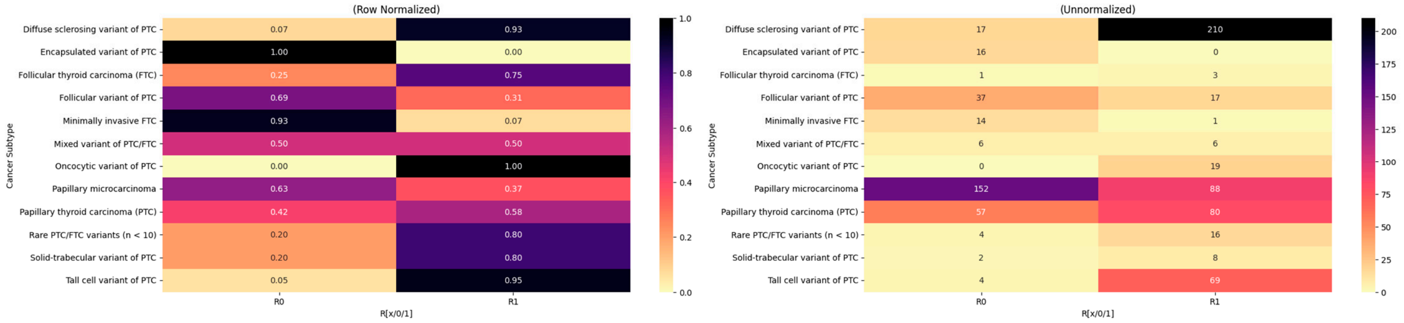

Figure S4. Heatmap Subtype - Margin Involvment

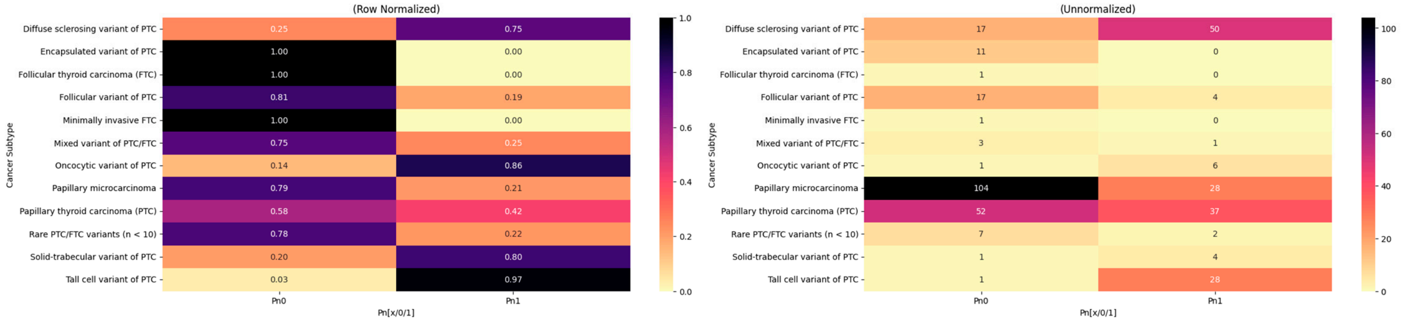

Figure S5. Heatmap Subtype - Perineural Invasion

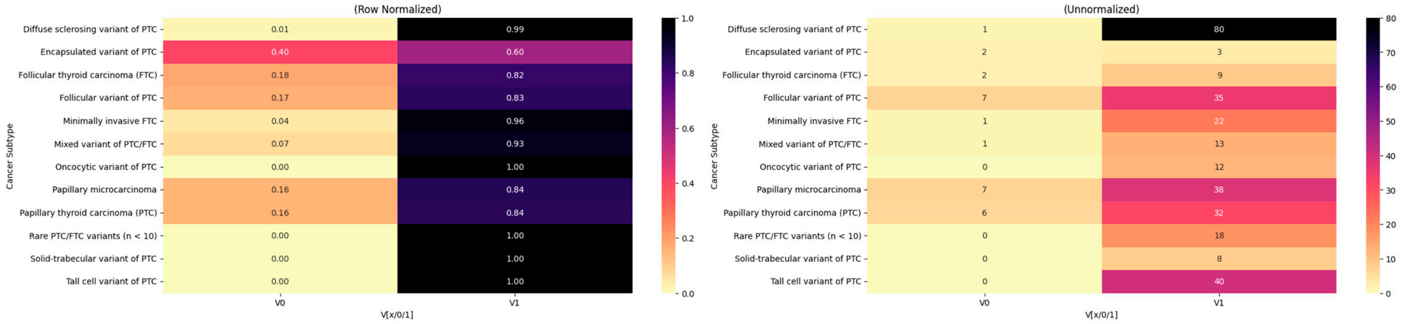

Figure S6. Heatmap Subtype - Vascular Invasion

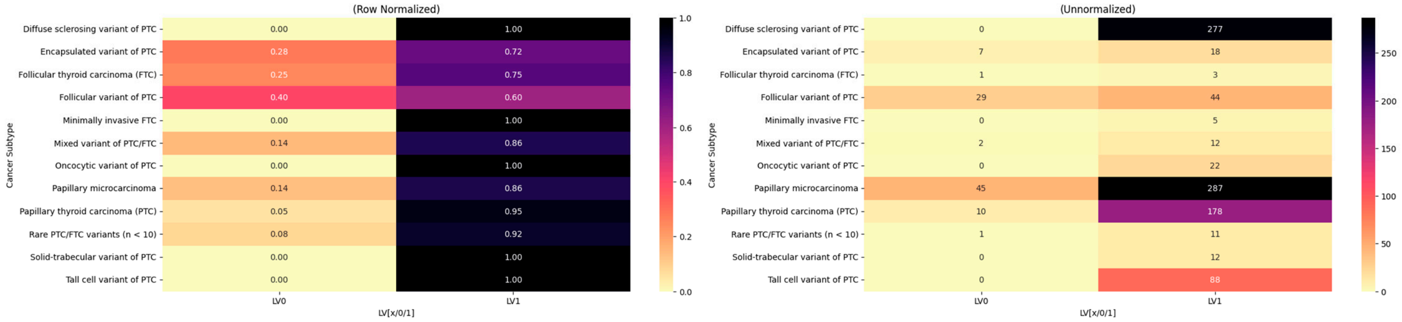

Figure S7. Heatmap Subtype - Lymphatic Vessel Extension
