# Supplementary material for: Exploratory Statistical Analyses of Clinical and Biochemical Factors for Differentiated Thyroid Cancer from a Romanian Cohort
Source: Cancers (Basel). 2026 Mar 23;18(6):1036. doi: 10.3390/cancers18061036 (PMC13025962; doi:10.3390/cancers18061036)
Supplement: Supplementary file 1 [file cancers-18-01036-s001.zip › File S3-heatmaps_unnormalized.pdf]

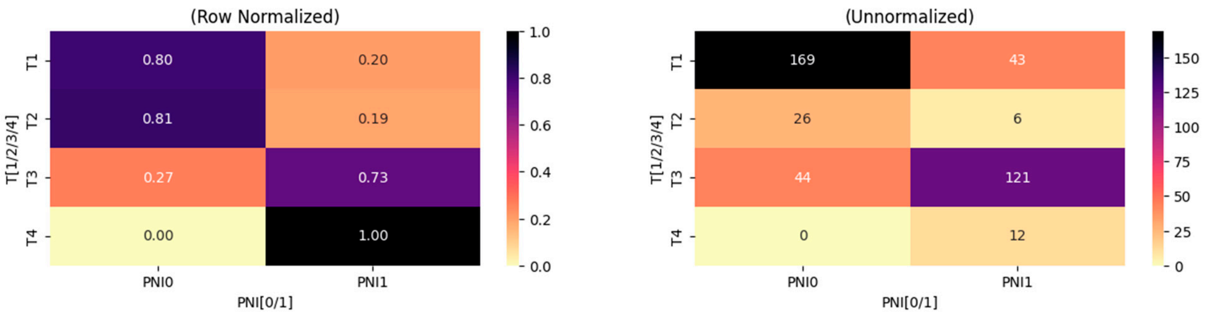

Figure S8. Primary tumor with perineural invasion

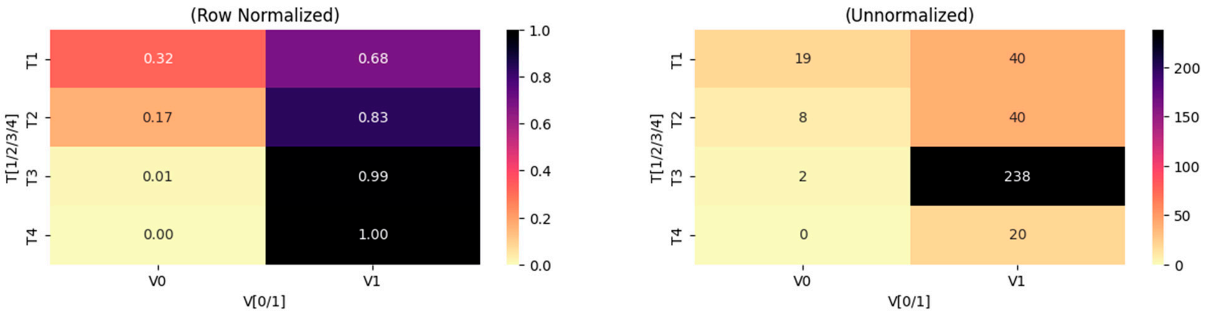

Figure S9. Primary tumor with vascular invasion

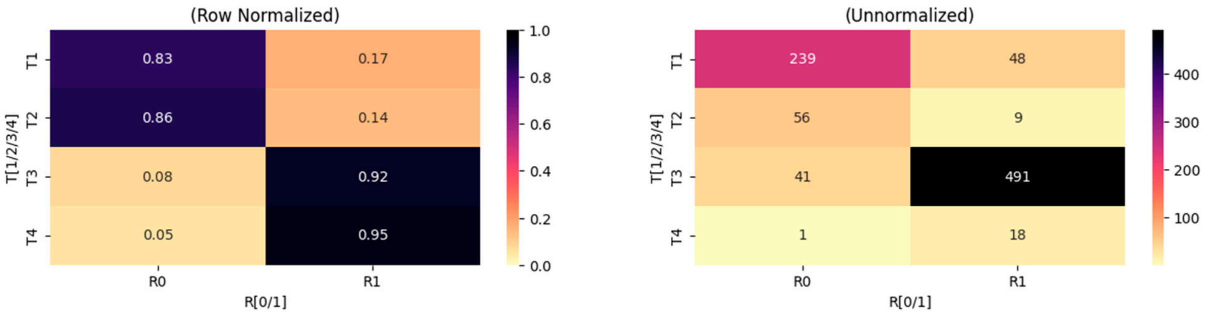

Figure S10. Primary tumor with margin involvement

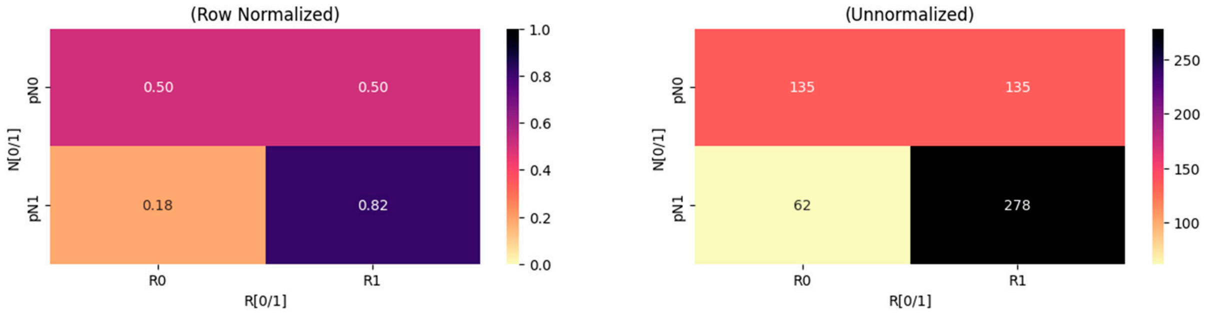

Figure S11. Metastases in the regional lymph nodes and margin involvment

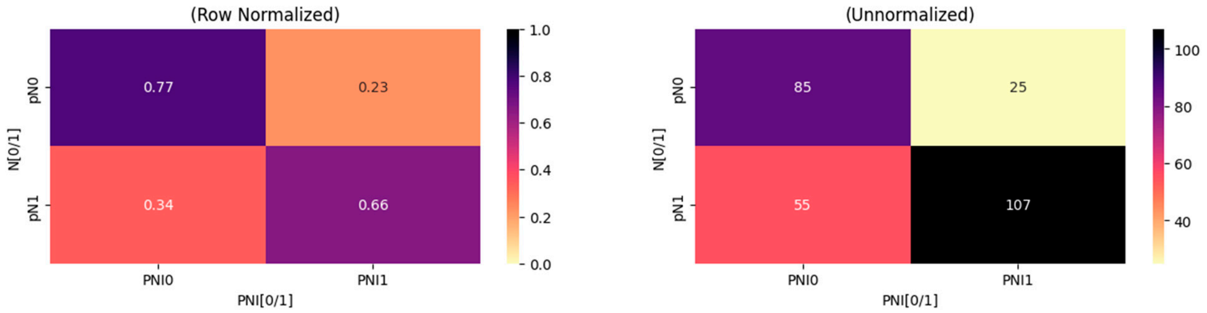

Figure S12. Metastases in the regional lymph nodes and perineural invasion

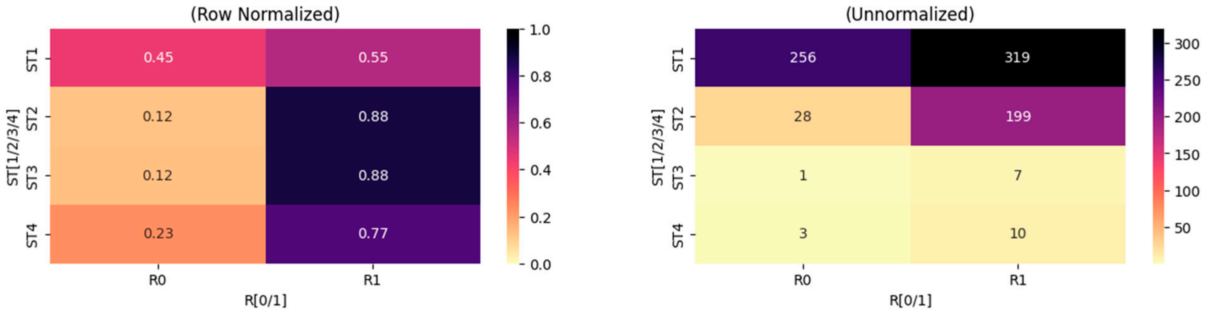

Figure S13. Tumor stage and margin involvment

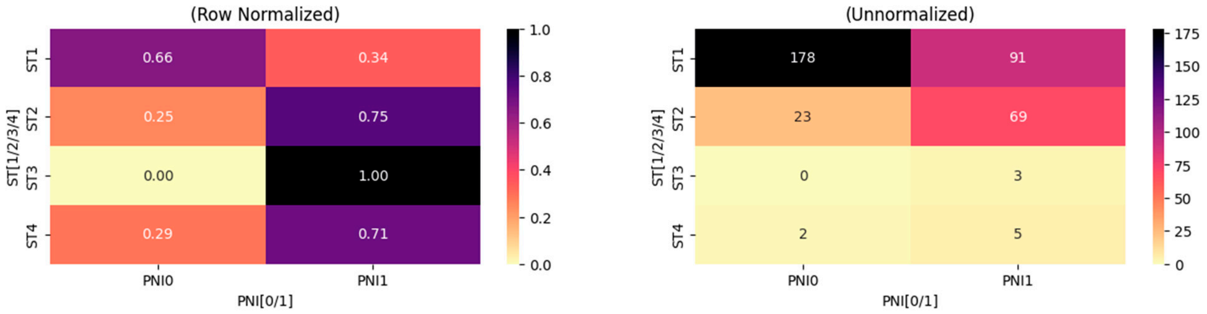

Figure S14. Tumor stage and perineural invasion

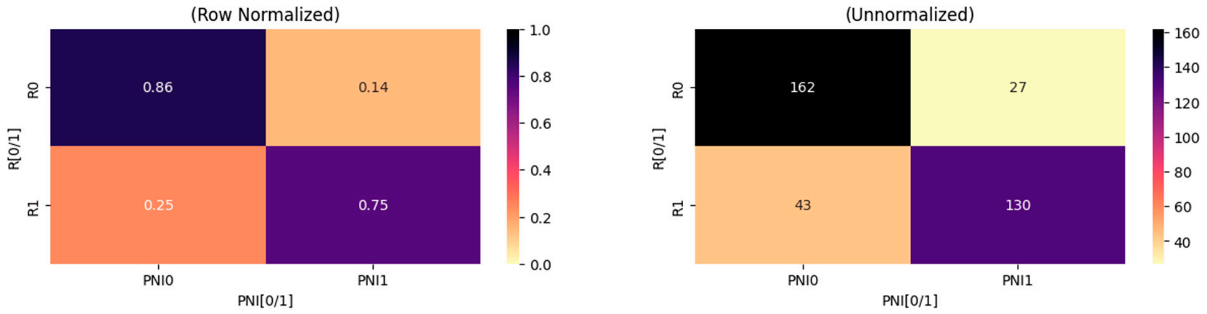

Figure S15. Perineural invasion and margin involvment

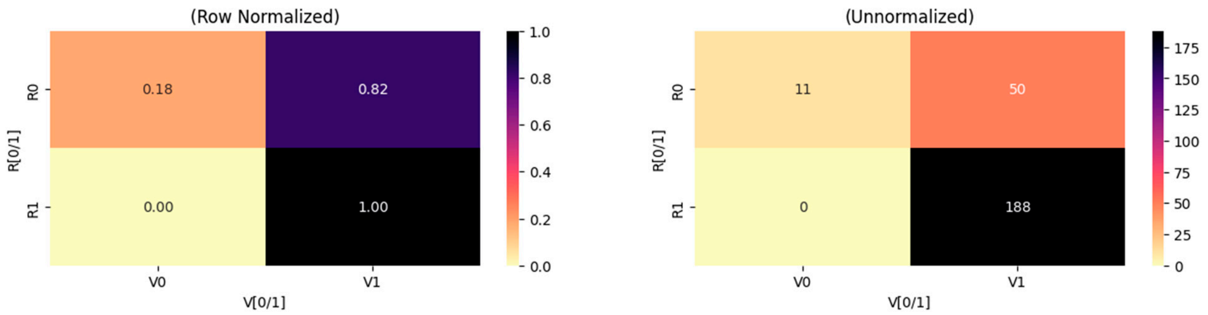

Figure S16. Vascular invasion and margin involvment

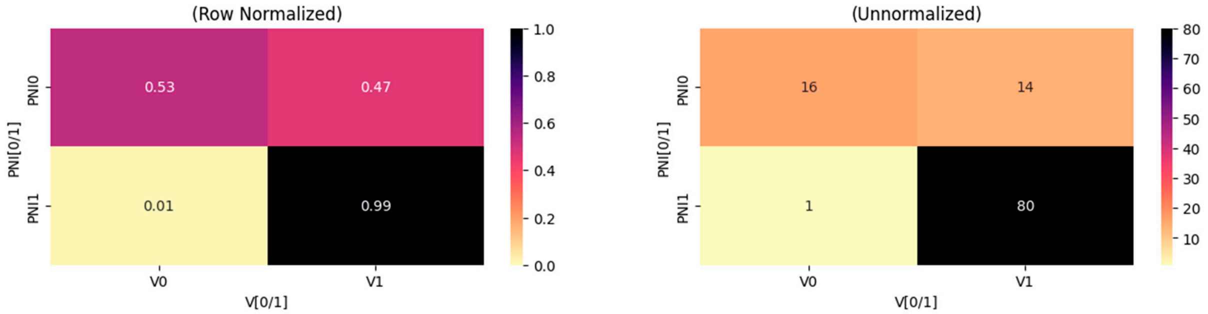

Figure S17. Perineural invasion and vascular invasion

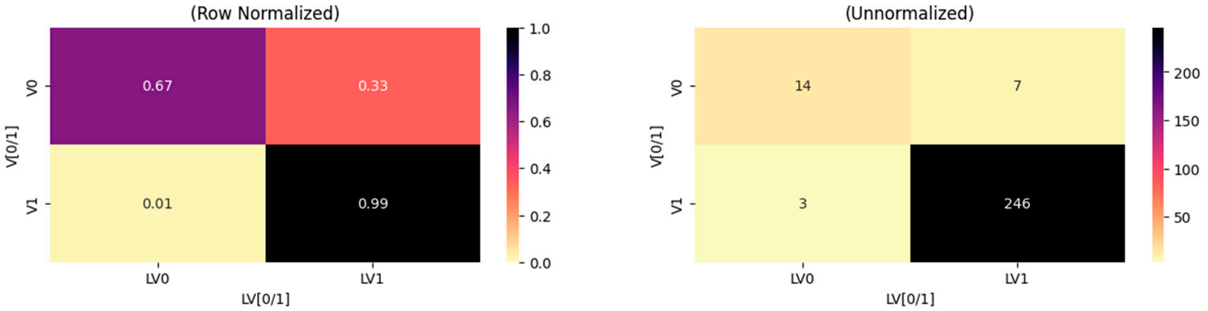

Figure S18. Vascular invasion and lymphatic vessel extension

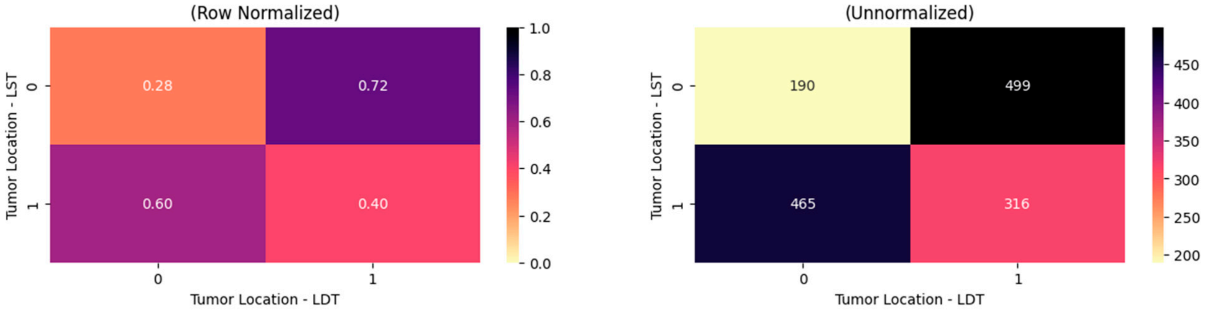

Figure S19. Left and right thyroid lobes (LST-left thyroid lobe, LDT-right thyroid lobe)
